# Current Understanding of the Physiopathology, Diagnosis and Therapeutic Approach to Alzheimer’s Disease

**DOI:** 10.3390/biomedicines9121910

**Published:** 2021-12-14

**Authors:** Victoria García-Morales, Anabel González-Acedo, Lucía Melguizo-Rodríguez, Teresa Pardo-Moreno, Víctor Javier Costela-Ruiz, María Montiel-Troya, Juan José Ramos-Rodríguez

**Affiliations:** 1 Department of Biomedicine, Biotechnology and Public Health, Physiology Area, Faculty of Medicine, University of Cádiz, 11003 Cádiz, Spain; victoria.garcia@uca.es; 2 Biomedical Group (BIO277), Department of Nursing, Faculty of Health Sciences, University of Granada, 18016 Granada, Spain; anabelglez@correo.ugr.es (A.G.-A.); vircoss@ugr.es (V.J.C.-R.); 3 Instituto de Investigación Biosanitaria, Ibs Granada, 18012 Granada, Spain; 4 Instituto Nacional de Gestión Sanitaria (INGESA), Primary Health Care, 51003 Ceuta, Spain; terepardo@correo.ugr.es; 5 Department of Nursing, Faculty of Health Sciences (Ceuta), University of Granada, 51001 Ceuta, Spain; mariamontiel@ugr.es; 6 Department of Physiology, Faculty of Health Sciences (Ceuta), University of Granada, 51001 Ceuta, Spain; juanjoseramos@go.ugr.es

**Keywords:** Alzheimer’s disease, senile plaques, β-amyloid protein, tau protein, diagnosis, biomarker, treatment, acetylcholinesterase inhibitors, immunotherapy

## Abstract

Alzheimer’s disease (AD) is the most common cause of dementia. It is characterized by cognitive decline and progressive memory loss. The aim of this review was to update the state of knowledge on the pathophysiological mechanisms, diagnostic methods and therapeutic approach to AD. Currently, the amyloid cascade hypothesis remains the leading theory in the pathophysiology of AD. This hypothesis states that amyloid-β (Aβ) deposition triggers a chemical cascade of events leading to the development of AD dementia. The antemortem diagnosis of AD is still based on clinical parameters. Diagnostic procedures in AD include fluid-based biomarkers such as those present in cerebrospinal fluid and plasma or diagnostic imaging methods. Currently, the therapeutic armory available focuses on symptom control and is based on four pillars: pharmacological treatment where acetylcholinesterase inhibitors stand out; pharmacological treatment under investigation which includes drugs focused on the control of Aβ pathology and tau hyperphosphorylation; treatment focusing on risk factors such as diabetes; or nonpharmacological treatments aimed at preventing development of the disease or treating symptoms through occupational therapy or psychological help. AD remains a largely unknown disease. Further research is needed to identify new biomarkers and therapies that can prevent progression of the pathology.

## 1. Introduction

Alzheimer’s disease (AD) is the leading cause of dementia in addition to being the most common neurodegenerative disease in the developed countries [1]. Anatomically, it is characterized by cerebral atrophy that especially affects the hippocampus and the entorhinal cortex, causing progressive memory loss and inability to carry out daily activities [2].

AD has been known since 1906 when psychiatrist and neurologist Alois Alzheimer described the findings after an autopsy performed on a 51-year-old patient after following the case of Auguste Deter who suffered from memory loss, inability to speak properly, disorientation and hallucinations between 1901 and 1906. Alois Alzheimer observed in the brain autopsy of this patient the presence of extraneuronal senile plaques (SP) and intraneuronal neurofibrillary tangles (NFTs), all accompanied by severe brain atrophy. These findings led him to believe that this was not a previously known dementia [3].

Only a century later, AD has become the leading cause of dementia, accounting for approximately 75% of all cases [4]. Currently, there are more than 50 million people affected by this dementia worldwide [5], of which 16.5 million are European, and an increase of more than 106.8 million cases is expected by 2050 [6]. These data are due to the fact that the main risk factor for the development of AD is age, and life expectancy has increased greatly over the last century. Only in Europe, life expectancy has increased by 35–40 years [7].

**Figure 1:**
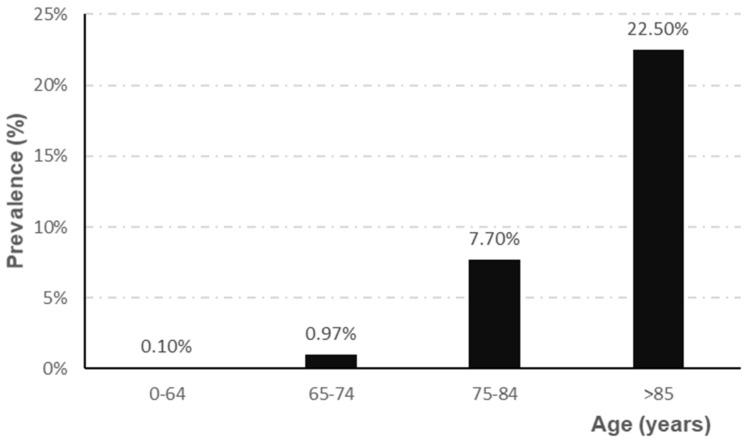
Evolution of the prevalence of AD according to age range (adapted from Garre-Olmo et al. [10]).

The prevalence of AD depends directly on the age range studied, showing a higher prevalence, for all ages, in women (5.1%) than in men (3.8%) [4]. Epidemiological studies indicate that prevalence increases sharply from the age of 65 [8,9,10], as shown in Figure 1.

The economic cost of treating and caring for AD patients has increased along with the incidence. In addition, the health care required by AD patients increases as the pathology evolves. In 2010, the cost of treating the leading cause of dementia was estimated at $604 billion worldwide. In just five years, this figure increased by 5%, reaching 818 billion dollars in 2015 [1]. Thus, while the average cost worldwide is between 3000ߝ6000$/year [11], in developed countries such as Spain, this figure is up to 30,000 euros/year [1]. On the other hand, AD causes significant social costs for the patient’s relatives. It is estimated that when a patient is diagnosed with AD, when cognitive impairment appears, the disease has already been present for about 10–12 years, evolving silently. After diagnosis, patients usually survive for between 5 and 8.5 years [10]. As the disease progresses, the care needs of AD patients increase, which also means an increase in the cost to the health care system and families [12]. In addition, it should be noted that during this stage, the quality of life of the patients worsens sharply, and they are declared to be the disabled who require health care or assistance throughout the day.

The aim of this review was to update the state of knowledge on the pathophysiological mechanisms, diagnostic methods and therapeutic approach to AD.

## 2. Results

### 2.1. Etiology of Alzheimer’s Disease

AD is a disease of unknown cause. However, it is believed that its etiology may be multifactorial, where risk factors play an important role. There is a small percentage of cases of AD of genetic origin, which constitutes less than 3% of all AD subtypes and has been called familial AD. Familial AD is characterized by earlier development (about 10–12 years earlier) compared to forms of idiopathic AD, known as sporadic AD [13]. Familial AD shows a dominant inheritance pattern [14]. The main mutations responsible for familial AD are in the *APP* gene, as well as in the proteolytic enzymes that generate the peptides Aβ, presenilin 1 and 2. There are other mutations that can increase the risk of developing this dementia. These include mutations in the *APOE* gene, where the *APOE4* variant predisposes to this dementia [15].

In addition to genetic factors, there are other factors that may contribute to the development of AD, such as high blood pressure [16,17], overweight and hypercholesterolemia [18,19], sedentary lifestyle, tobacco use, low level of education [20], diabetes mellitus or hyperinsulinemia [21,22,23]. All these factors together are involved in the development of 33.3% of AD cases [20].

### 2.2. Clinical Stages of Alzheimer’s Disease

The global and functional clinical stages of AD are summarized as follows. There is an initial phase, commonly known as the prodromal phase, whose main characteristic is a mild cognitive impairment, i.e., with subtle memory loss, which often goes unnoticed [24]. As the disease progresses, different cortical functions are altered, causing difficulties in the development of basic activities of daily living [25]. When the disease reaches the final stages, patients with AD become totally dependent, compromising their lives and causing significant changes for the relatives, who become caregivers [26].

However, there is a long transition period between the appearance of alterations at the brain level and the presentation of the first clinical symptoms [24]. The severity of AD according to its clinical symptomatology can be classified as mild, moderate or severe. As the disease worsens, so do the symptoms, which can range from mild cognitive impairment, increased memory loss, personality variations, problems in carrying out everyday tasks to confusion, psychomotor difficulty, loss of speech and ultimately death of the patient [2].

### 2.3. Neuropathological Features

Currently, AD has no accurate diagnosis. Its definite diagnosis is still limited to post-mortem examination of brain tissue. In order to diagnose this pathology, its three defining characteristics must be present [27]: amyloid-β pathology, tau pathology and neuroinflammation, neuronal death and brain atrophy. Currently, the amyloid cascade hypothesis continues to be the explanatory model for describing the onset and histopathological evolution of AD. This hypothesis states that amyloid-β (Aβ) deposition in the brain parenchyma triggers a chemical cascade of events leading to the development of AD dementia.

#### 2.3.1. Amyloid-β Pathology (Aβ Pathology)

SP are characteristic alterations of AD very frequent in the brain of patients with dementia and possibly the origin of denervation of the disease.

**Figure 2:**
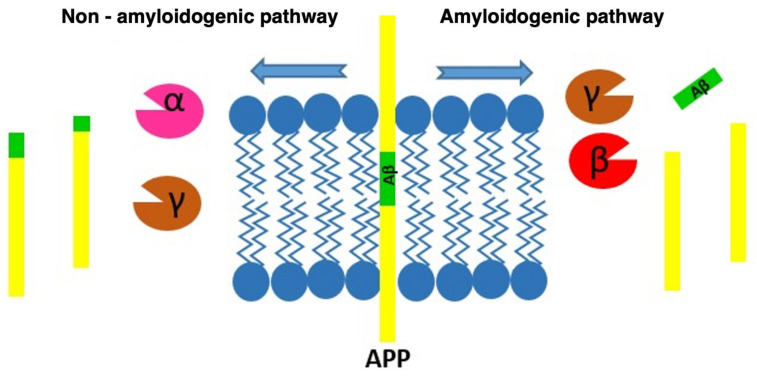
Diagram of the processing routes by which the APP can be degraded, showing how the β-amyloid peptide is produced.

SP are the result of the progressive accumulation of parenchymal Aβ [3]. Aβ is a peptide of between 39–43 amino acids derived from progressive processing of the Aβ precursor protein (APP) by the β- and γ-secretase complexes, where presenilins would be the catalytic component [28]. APP is one of the proteins found in greater proportion in the central nervous system [29]. Aβ is derived from the proteolytic breakdown of the APP protein which, when processed by the β- and γ-secretases, results in three products, among which is the Aβ which promotes the formation of SP (Figure 2).

In healthy patients, in contrast to what occurs in AD patients, the non-amyloidogenic route is favored, which is mediated by the γ- and α-secretases and whose residues do not include Aβ. Among the possible isoforms of Aβ, Aβ 40 and Aβ 42 are the most common, and Aβ 42 is the most fibrillogenic form. Monomers from Aβ are released into the extraneuronal space, although they can easily enter the neuronal cytoplasm. The monomers from Aβ tend to agglutinate forming dimers, trimers and major structures that eventually form the SP that are deposited in the extraneuronal space. These extracellular deposits are mostly constituted by two isotherms of Aβ, the Aβ 40 and the Aβ 42 [30]. The peptide Aβ42 is considered the most toxic [29], and several studies suggest that high levels of this peptide cause a disruption of synapses and neuronal degeneration. This represents an important element in the development of AD [31]. In addition, it should be noted that before the appearance of SP, an accumulation and increase in peptides Aβ42 associated with aging is observed [32].

At the synaptic level, Aβ makes synaptic transmission difficult. The monomers in Aβ inhibit the neurotransmitter receptors and prevent the sodium–potassium pump from working correctly at the synaptic level, making electrical and chemical transmission difficult and blocking the correct interneuronal connection [33]. In addition, this peptide promotes internalization of the channels associated with neurotransmitter receptors. This situation, when maintained over time, causes a synaptic dysfunction and subsequent loss of neuronal synapses [34]. In fact, high levels of Aβ have been associated with progressive loss of synaptic density and the appearance of neuritic dystrophies. The loss of synaptic functionality and subsequent destruction of synaptic connections by the action of the Aβ and tau pathology in AD [35] is the neuropathological feature that is best related to cognitive impairment [36]. This suggests that synaptic density and its correct functioning are the basis for the correct development and performance of cognitive functions.

The peptides Aβ in their different aggregation states and compact SP are neurotoxic in both AD and experimental models [37] and have been associated with synaptic loss and neuritic dystrophies [38,39,40]. Compact SP have also been associated with abnormal curvature of neighboring neurites [41,42,43] and may alter cortical synaptic integration [44]. SP usually appear in the parietal cortex and then spread to the rest of the cortex [45]. It is important to highlight that the presence of deposits of Aβ are not totally related to neurodegenerative processes since during their evolution no brain atrophy is detected. In this sense, tau pathology presents a stronger involvement in the processes of neuronal death and brain atrophy as described below [46].

Peptide Aβ40 is the isoform that mostly tends to accumulate in the walls of cerebral and leptomeningeal arteries [47], resulting in cerebral amyloid angiopathy (CAA), a subtype of Aβ pathology frequently observed in people who suffer from AD [48]. Up to 90% of AD patients present with this alteration [49]. However, CAA can also appear in patients without AD [50], being the main exponent of other dementia forms, such as vascular dementia. CAA causes a decrease in blood perfusion through the affected vessels and a higher incidence of cerebral ischemia [51]. On the other hand, the clearance of excess monomers from Aβ can be carried out through the excretion of these peptides into blood vessels. In AD, the clearance of vascular Aβ is compromised and the accumulation of deposits in the form of CAA on vessels is promoted [52].

Another route of Aβ monomer degradation is through neuronal enzymes. Among them, enzymes such as neprilysin and insulin-degrading enzyme stand out for their activity [53]. Neprilysin is a transmembrane peptide capable of degrading Aβ dimers. It is synthesized by neurons; however, no increased neprilysin production has been detected in Aβ pathology. In this sense, there are studies that point out that enhancing the production of this enzyme increases neuronal protection and survival in AD [54].

On the other hand, the insulin-degrading enzyme can degrade insulin and Aβ monomers. It is a protein synthesized by neurons and can be found freely in the cytoplasm or in the extracellular space. Its production is altered in AD patients [55]. Its role as a link between AD and diabetes mellitus has been proposed, due to its double degrading action on Aβ and insulin. In patients with AD and type 2 diabetes mellitus there is overproduction of both peptides, so there is a competition for both to be degraded. This fact would cause, secondarily, the accumulation of both peptides at the brain level [56]. Stimulation of the production of the insulin-degrading enzyme decreases the levels of Aβ and the formation of SP [57] which could be used as a therapeutic alternative in the treatment of AD.

The accumulation of Aβ in the medial parietal cortex constitutes the first stage in AD, although the concentration of NFTs in the medial temporal lobe occurs before the accumulation of Aβ in people without AD [45]. It is important to note that Aβ is not entirely related to neurodegeneration, with the tau protein being more involved in brain atrophy [46].

#### 2.3.2. Tau Pathology

NFTs are intraneuronal deposits mainly composed of hyperphosphorylated tau protein which form filamentous aggregates in neuronal somas and proximal dendrites [58,59].

**Figure 3:**
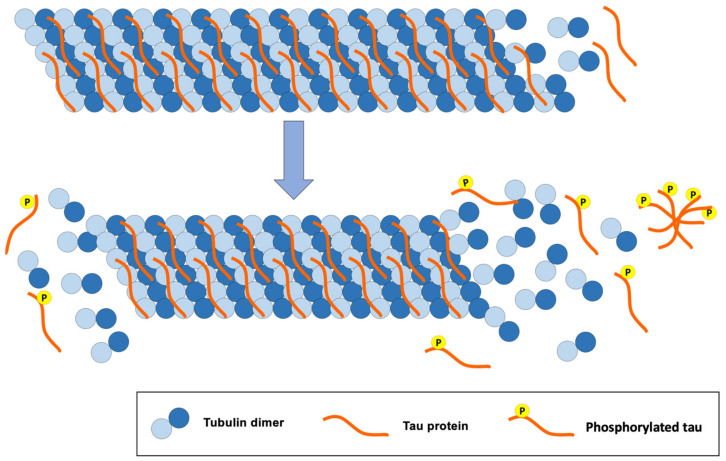
Consequences of tau protein hyperphosphorylation for the structure of tubulin microtubules, a classic pathology of AD.

The tau protein is mostly found in the central nervous system and in the peripheral nervous system. It is a small protein that is attached to microtubules, offering stability when interacting with them (Figure 3). Tau is a protein found abundantly in the axons where microtubules predominate. These microtubules play an important role in the structure of neurons, axon transport and synaptic plasticity of neurons [29].

The structure of microtubules is modified when inadequate phosphorylation of the tau protein occurs. Likewise, hyperphosphorylation of this protein disrupts the synapses between neurons, causing cellular alterations that lead to the loss of synapses, neuronal ramifications and neuronal death [29].

Hyperphosphorylation of the tau protein usually begins in the entorhinal cortex (anterolateral region) and then progresses to the hippocampus. The entorhinal cortex is divided into two areas, the posteromedial region and the anterolateral region. Each is related to a type of memory. The posteromedial subregion is involved in spatial memory and the anterolateral subregion is related to the episodic memory [45]. The appearance of NFTs is one of the pathological events observed in the most developed stages of AD. Their presence is closely related to the clinical evolution of the AD patient. In fact, it is the neuropathological characteristic that best correlates with cognitive impairment [60,61]. Phosphorylated tau proteins promote phosphorylation of other tau proteins spreading within a neuron to other neurons through neuronal synapses [45].

As mentioned above, there is a period of approximately 10–12 years between the development of histological pathologies in the brain and the establishment of clinical manifestations. During this time, the Aβ peptides and the tau protein accumulates, forming SP and NFTs, respectively [45]. However, while SP are proportionally distributed among the different regions affected in AD [62], NFTs are mainly found in the entorhinal cortex and hippocampus, two structures that play an important role in memory [45]. It should be noted that the evolution of intraneuronal deposition of NFTs is closely related to neuronal loss and brain atrophy. In this sense, it is known that the accumulation of NFTs induces neuronal death which leads to the formation of charged sacks of this hyperphosphorylated protein known as neuronal ghosts [63].

In addition to Aβ pathology and tau pathology, neuroinflammation also plays a major role in neurodegenerative processes [64]. Inflammation is a protective response, but its chronification can cause tissue damage [65]. At the central level, microglial cells are involved in the inflammatory response. In this sense, brain inflammation is another characteristic feature of AD, with alterations observed in the morphology and distribution of the microglia, which increases the expression of cytokines and proinflammatory mediators in AD [66]. The cells of the microglia have phagocytic functions acting as macrophages at the brain level with the capacity to eliminate toxic elements from the environment and promote tissue inflammation [67]. Thus, microglia represent the first defense system of the central nervous system. Since the first events in the physiopathology of AD, when the levels of Aβ increase without necessarily forming SP, activation of the microglia has been observed [23].

Later, when SP appear, microglial cells move toward the plaques and surround them within the first 24 h. Although they manage to surround and stop their growth, they do not appear to have phagocytic capacity against the plaques, which persist throughout the patient’s life [68]. This event causes microglia to remain active from the beginning of AD, establishing a pattern of proinflammatory cytokines that end up generating an environment of neurotoxic oxidative stress, which contributes to and correlates with the neuronal death observed in AD [69]. In addition, a proinflammatory state maintained over time reduces the capacity to eliminate Aβ, accelerating and worsening the evolution of AD [66,70,71].

Neuronal death and thus brain atrophy is the latest event in the pathogenesis of AD. It seems that neuronal death is not determined by a single factor, but rather is the result of the sum of different factors, among which Aβ pathology, tau pathology and neuroinflammation stand out [64].

All this pathogenesis which ends with neuronal death responds to a time sequence. Thus, the first outstanding event is usually the overproduction of Aβ in its different isoforms with its neurotoxic effects, including those mediated by the toxic effects of neuroinflammation. The accumulation of NFTs is usually the second event that precedes neuronal death, which eventually leads to cerebral atrophy in AD patients, mainly affecting the hippocampus and secondarily the cerebral cortex [31].

### 2.4. Diagnosis of AD

Despite the numerous advances that have been made in the field of medicine, the antemortem diagnosis of AD continues to be a challenge for the scientific community since it is still based on clinical parameters, which can make it difficult to establish a differential diagnosis with other neurodegenerative pathologies [72]. However, the clinically based diagnosis of AD (typical or atypical presentations) is probabilistic [73]. In this sense, it is necessary to define the biological characteristics of this disease in order to establish clear indicators that reflect the underlying neurological alterations [74].

New diagnostic methods are currently gaining prominence, including fluid and image-based biomarkers which have been incorporated in diagnostic criteria and recommendations for AD [75,76]. The main fluid-based biomarkers are those present in cerebrospinal fluid (CSF) and plasma [77]. In CSF, the molecular markers that have been shown to be most clinically useful are Aβ42 and phosphorylated tau. Thus, it has been observed that as humans age, Aβ42 levels decrease and phosphorylated tau levels increase, reflecting possible deposition in the brain leading to SP and the formation of NFTs, respectively [78]. Although this is a widely accepted diagnostic method, sampling requires lumbar puncture which is a relatively invasive procedure, justifying the search for other less aggressive methods such as plasma biomarkers [79]. These are obtained through a metabolomic and lipidomic analysis that provides diagnosis with a reliability of more than 90% even three years before the onset of the first symptoms. In this sense, plasma biomarkers such as phosphorylated tau 181, phosphorylated tau 127, P231, Total tau and Nfl have demonstrated their potential in the diagnosis of AD, ptau127 being the marker with the highest diagnostic accuracy [80,81,82,83]. Likewise, it has been shown that lower levels of serotonin, phenylalanine, lysine, phosphatidylcholine, acylcarnitine and proline are associated with greater progression of AD [84].

On the other hand, biomarkers based on diagnostic imaging methods provide information about histological abnormalities compatible with AD. Among these markers are positron tomography (PET) and high-resolution magnetic resonance imaging (MRI) [85,86,87,88]. PET is a diagnostic method that elucidates the processes involved in cellular metabolism and can map the specific proteins involved in the disease. Thus, with the help of radiotracers such as 18F-THK523 or 11C-PBB3, it is possible to identify the accumulation of phosphorylated tau and Aβ and perform quantitative monitoring of AD [89,90]. As for MRI, this technique deals with the identification of anatomical changes in the brain in a precise and noninvasive way either through structural or functional MRI. Structural MRI, through a volumetric analysis of tissues, explores possible cortical thinning as well as a decrease in the volume of gray matter in the hippocampus [91] while functional MRI analyzes brain connectivity from the anatomical point of view, measuring the diffusion of protons within the organism. Thus, some studies have described a relationship between decreased connectivity and increased symptomatology related to cognitive impairment [31].

In addition to the diagnostic procedures described above, there are other noninvasive techniques that are currently being studied, such as the identification of Aβ42 and phosphorylated tau in saliva [92] or the study of molecules that act as epigenetic factors (miRNA) [93]. These advances would not only facilitate early diagnosis, but could also contribute to the staging of the disease and the establishment of prognosis and possible treatment [77]. However, no conclusive results have yet been obtained to clarify their clinical utility, which reinforces the need to develop new studies that would allow their validation and transfer to practice.

### 2.5. Alzheimer’s Disease Treatment and Management

#### 2.5.1. Currently Available Pharmacological Treatment

**Figure 4:**
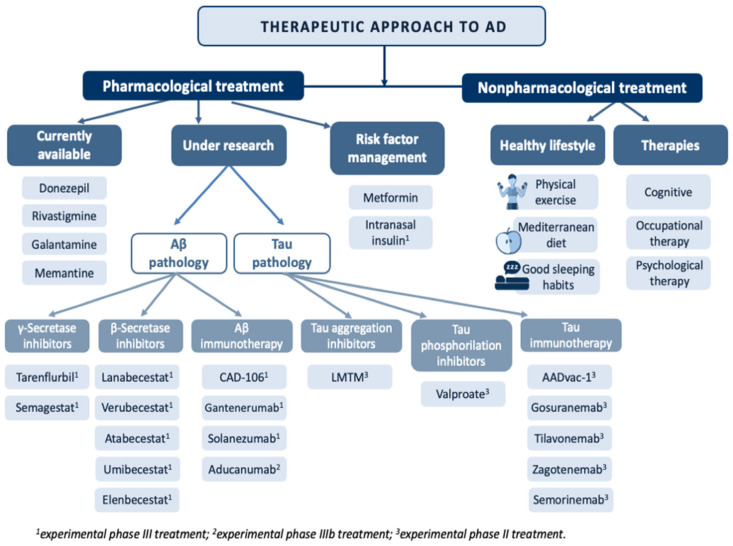
Overview of the main treatments in the therapeutic approach to Alzheimer’s disease.

A curative treatment of AD remains to be discovered. Currently, the therapeutic armory available focuses on symptom control in milder cases of the disease [94,95,96] (Figure 4).

Among the pharmacological alternatives available, acetylcholinesterase inhibitors (AChEIs) stand out [97]. The mechanism of action of AChEIs is based on the inhibition of acetylcholinesterase (AChE) activity, which degrades acetylcholine (ACh), increasing its availability, which is reduced in AD. Drugs available in this group for the treatment of AD include:

- Donepezil: Approved in 1996 [98], donepezil is the pharmacological treatment of choice for AD. This drug acts by reversibly binding to AChE, inhibiting ACh hydrolysis, resulting in increased bioavailability of this neurotransmitter at neuronal synapses. Its use has been shown to slow cognitive decline and improve behavior in people with AD [97].
- Rivastigmine: This treatment was introduced in 2000 and is indicated for mild and moderate AD. It should be noted that unlike the other AChEIs, rivastigmine is also used to treat dementia associated with Parkinson’s disease [98]. This drug exerts its effect by binding to and inhibiting AChE, increasing ACh levels [97]. The use of rivastigmine in transdermal patches has been shown to be beneficial in AD patients with swallowing problems while decreasing the side effects seen with lower doses in the pill form [99].
- Galantamine: This is a competitive AChE inhibitor approved in 2001 [98]. Its use in mild and moderate AD has been associated with positive evolution of behavior, cognitive performance and development of basic activities of daily living [97]. In addition, it has been observed that galantamine is able to cross the blood–brain barrier more quickly, affecting brain areas such as the hippocampus for an extended period of time (5–7 h) [100].
- Memantine: The latest drug approved for use in moderate and severe AD as monotherapy or in combination with other therapies [101]. It is generally well-tolerated and safe as it exerts its effect without altering neuronal synapses [97]. Memantine treatment has been shown to improve cognitive impairment and general condition in AD patients [101].

In relation to the use of these drugs, it is important to advise the performance of an electrocardiogram before initiating treatment with AChEI due to the risk of developing sick sinus syndrome as well as other cardiac abnormalities associated with the electrical conduction of the heart [102].

#### 2.5.2. Pharmacological Treatment under Investigation

The lack of effective treatments to prevent and slow the progression of AD, together with the increasing demand for new drugs, has motivated the search for therapeutic alternatives aimed at controlling the pathophysiological mechanisms underlying this disease, including Aβ pathology and hyperphosphorylation of the tau protein [103].

Treatments affecting Aβ pathology focus on three therapeutic targets. The first is aimed at reducing the overproduction of Aβ42 through γ-secretase inhibitors, β-secretase inhibitors or α-secretase enhancers. The second therapeutic target focuses on reducing the Aβ load in SPs by using aggregation inhibitors or drugs that interact with the metals that are deposited on them. The third therapeutic target is aimed at boosting Aβ clearance by active or passive immunotherapy [104].

With regard to γ-secretase inhibitors, it is important to highlight the role of tarenflurbil and semagestat. Both drugs have been studied in several phase II clinical trials, resulting in a marked slowing of cognitive decline and a reduction in Aβ40 levels, respectively. However, the poor effectiveness of tarenflurbil and the side effects caused by semagestat have led to the interruption of phase III clinical trials [105].

Several β-secretase inhibitors, such as lanabecestat, verubecestat, atabecestat, umibecestat and elenbecestat, have been successful in reducing Aβ levels in CSF. However, the trials were stopped in phase III due to cognitive and functional worsening in patients treated with these drugs, as well as the development of adverse effects [106].

Finally, Aβ clearance through immunotherapy is one of the latest therapeutic approaches being considered. In this line, there are numerous clinical trials of drugs that have reached phase III, including CAD106, gantenerumab, solanezumab and aducanumab [104]. Among them, CAD106 and aducanumab stand out due to their advanced experimental phase. CAD106 has been successful in reducing Aβ accumulation, proving to be safe and well-tolerated in patients with mild AD [107]. Aducanumab has undergone two phase IIIb clinical trials, with a small improvement in patients in one of these trials [108]. Despite controversy, in mid-2021, aducanumab was approved in the United States by the Food and Drug Administration for use in patients with mild AD. The tolerability and safety of this treatment is currently being evaluated. In Europe and Japan, approval is still under evaluation [109].

Due to contradictory results of the many drug trials targeting Aβ pathology, recent studies have focused on the therapeutic approach to tau pathology [110]. The proposed therapies aim to inhibit abnormal hyperphosphorylation and reduce tau protein accumulation, as well as contribute to the development of active and passive immunotherapies [111]. Among the drugs targeting inhibition of phosphorylated tau aggregation, a methylene blue derivative called leuco-methylthioninium bis(hydromethanesulfonate) (LMTM) stands out for its good absorption and tolerance [112]. This treatment is still in phase III testing in mild AD, although early results seem to indicate little success in reducing cognitive decline [112]. Another pharmacological approach to reduce tau pathology is through inhibition of the kinases that promote tau phosphorylation. In this regard, inhibitors targeting glycogen synthase kinase 3 (GSK3β) are the most widely used, most notably valproate [113]. This drug has been successful in inhibiting GSK3β kinase in animal models, with promising results. However, a phase III clinical trial showed that it has neurotoxic properties that promote progression of brain atrophy and accelerate cognitive impairment, discarding it as a therapeutic alternative [114].

The newest pharmacological approach in the treatment of tau pathology is immunotherapy. As in Aβ pathology, immunotherapies have been developed with the aim of generating an autoimmune response against hyperphosphorylated tau protein. Among the immunotherapy-based treatments, several drugs have reached phase II, such as gosuranemab, tilavonemab, zagotenemab and semorinemab. Although positive results have been reported in experimental animals, in humans, they have only been shown to slightly decrease levels of the phosphorylated tau protein, without showing cognitive improvement [113]. Only the use of AADvac-1 in patients with mild to moderate AD has reached phase II, showing a reduction in hyperphosphorylated tau in CSF and a slowing of cognitive decline [115].

#### 2.5.3. Risk Factor Management

AD is a multifactorial pathology where risk factors play an important role in the pathophysiology of the disease. One of the most important is type 2 diabetes mellitus as insulin resistance in the central nervous system has been observed in AD patients. Therefore, increasing the availability or sensitivity to insulin at the brain level could be a possible therapeutic alternative for the management of AD patients [116]. In fact, administration of oral antidiabetic drugs, and more specifically of metformin, has been shown to have a neuroprotective, anti-inflammatory and antioxidant action [117]. In addition, one of the therapeutic approaches under study is the administration of intranasal insulin [118]. In addition to diabetes, some cardiovascular events such as stroke, hypertension, hypercholesterolemia, heart failure or atrial fibrillation and other risk factors such as high homocysteine or smoking have been associated with the development of AD [119,120,121,122,123,124]. Thus, the design of preventive strategies that allow early diagnosis and treatment of cardiovascular risk factors could contribute significantly to the prevention of AD in the elderly.

#### 2.5.4. Nonpharmacological Treatment

In addition to all the treatments mentioned so far, in the therapeutic approach to AD, it is worth highlighting the role of different health professionals such as occupational therapists and psychologists. Occupational therapy represents an important part of the treatment as it increases the autonomy of these patients, allowing the development of activities of daily living through cognitive and behavioral exercises [125]. Psychological therapy is fundamental in AD patients as it provides them with the necessary tools to deal with processes such as depression or anxiety, two very common clinical manifestations in AD patients that have important repercussions at both the cognitive and functional levels [126]. Finally, it is important to emphasize that the best treatment for any disease lies in a good prevention strategy. In this regard, adopting a healthy lifestyle that includes physical exercise [127], a Mediterranean diet and a good sleep habit has proven to be one of the effective strategies as it has been associated with a reduction in cognitive decline and a lower risk of developing AD [128]. Cognitive therapy has also been positioned as the non-pharmacological treatment with the best results in the prevention of AD. This therapy is characterized by the development of cognitive stimulation and training exercises.

## 3. Conclusions

AD remains the leading cause of dementia in addition to being the most common neurodegenerative disease in developed countries affecting more than 50 million people worldwide. Despite these figures, AD remains a largely unknown disease as the evidence on the pathophysiological mechanisms underlying AD remains controversial. Currently, the amyloid cascade hypothesis remains the leading theory in the pathophysiology of AD. Likewise, the antemortem diagnosis of AD is still based on clinical parameters or fluid-based biomarkers or diagnostic imaging methods. The therapeutic armory available focuses on symptom control, where we can distinguish between four main strategies: pharmacological treatment where acetylcholinesterase inhibitors stand out; pharmacological treatment under investigation which includes drugs focused on the control of Aβ pathology and tau hyperphosphorylation; treatment focusing on risk factors; or nonpharmacological treatments aimed at preventing the development of the disease or treating symptoms through occupational therapy or psychological help. This lack of in-depth knowledge of the disease both at the pathophysiological level and with regard to diagnosis and treatment justifies the search for potential biomarkers that can contribute to determining the stage of the disease, especially in initial stages, as well as new pharmacological and nonpharmacological treatments that would enable an early therapeutic approach to the pathology by controlling its progression and improving the quality of life of AD patients, their caregivers and relatives.
